# Cracking the Olfactory Code

**DOI:** 10.1371/journal.pbio.0030122

**Published:** 2005-02-22

For Proust, a taste of cookie was enough to trigger vivid recollections of his childhood, the first of a long string of reveries that he fashioned into his famous memoir *Remembrance of Things Past*. For many animals, too, tastes and smells are evocative and play a crucial role in finding food, allowing them to build on past successes and to learn how to find their next meal.

To locate blooming flowers, for example, honeybees rely heavily on scent. They can associate a whiff of an aldehyde, say, with a nectar-filled orchid. Then later they'll seek out the same or similar scents. To succeed in the wild, they must be able to distinguish relevant scents at varying concentrations, and within complex milieus of other scents. But to find food in varied conditions and adapt to new situations, they also have to generalize from past experience.

Through both physiological and behavioral studies, scientists have investigated the response to smell in a wide range of organisms and have suggested that two key properties of scent-inducing chemicals are the functional class, such as alcohol or aldehyde, and the carbon-chain length. Bees trained to associate a particular chemical with a reward, for example, can then generalize to some extent to other chemicals with the same functional groups or similar carbon-chain lengths. In these situations, bees are surprisingly consistent in both in their behavior (extending their proboscis to an odor previously associated with food) and in their brains (brain activity in smell-processing centers). Each set of data, behavioral and neural, can be thought of as a “code” underlying the bee's response: present a scent, and a bee's brain and body will tend to react in a certain way.[Fig pbio-0030122-g001]

**Figure pbio-0030122-g001:**
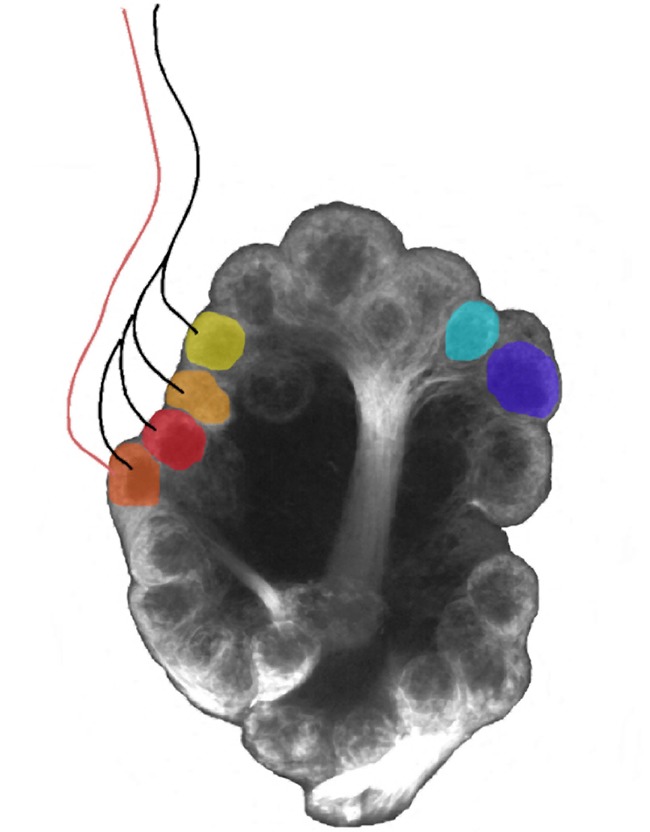
Linking smellperception and neural activity in the bee (Image: Axel Brockmann)

A new study of smell perception in honeybees (Apis mellifera) published in *PLoS Biology* gives a more comprehensive picture of how bees react to a suite of scents and also shows a remarkable correspondence between the codes for the insects' behavior and brain activity. The researchers, led by Martin Giurfa, first trained bees to associate a specific chemical, such as the alcohol 1-nonanol, with a sucrose reward. Then the researchers tested the bees' response to a set of other chemicals, varying in carbon-chain length from six to nine, and with four different functional groups: aldehydes, ketones, and primary and secondary alcohols.

By watching how often the bees generalized—that is, how often they responded positively to a particular scent when they'd been trained on another—the researchers could assign perceptual “distances” between pairs of chemicals. Drawing together all these distances, they created a preliminary map of the bees' “perceptual space,” similar to how surveyors measure distances between landmarks to map a landscape. From this comparison they found, for example, that the bees generalized more by functional group than by carbon-chain length.

Previously, Giovanni Galizia's group, which works closely with Giurfa's group, had recorded bees' brain responses to the same pairs of scents, assigning distances within centers of activity for each scent. Giurfa's team compared these two sets of data and found that the perceptual and neural distances correlated well, which suggests there's a species-specific code that ties together the insects' brain and behavior.

The brain recordings covered only a quarter of the bees' main smell-processing center, the antennal lobe. Future studies with new methods of microscopy that visualize more of the brain and which focus on the olfactory message sent by the antennal lobe to higher-order brain centers should only improve our ability to investigate the correlations between brain and behavior, the authors say. Such studies would go even further toward cracking the codes underlying animals' perception and memory.

